# Chloroquine reduces arylsulphatase B activity and increases chondroitin-4-sulphate: implications for mechanisms of action and resistance

**DOI:** 10.1186/1475-2875-8-303

**Published:** 2009-12-17

**Authors:** Sumit Bhattacharyya, Kemal Solakyildirim, Zhenqing Zhang, Robert J Linhardt, Joanne K Tobacman

**Affiliations:** 1 Department of Medicine, University of Illinois at Chicago, 840 S. Wood St., CSN 440 M/C 718 Chicago, IL 60612, USA; 2 Jesse Brown VA Medical Center, Chicago, IL, USA; 3 Department of Chemistry and Chemical Biology, Rensselaer Polytechnic Institute, Troy, NY, USA

## Abstract

**Background:**

The receptors for adhesion of *Plasmodium falciparum*-infected red blood cells (RBC) in the placenta have been identified as chondroitin-4-sulphate (C4S) proteoglycans, and the more sulphate-rich chondroitin oligosaccharides have been reported to inhibit adhesion. Since the anti-malarial drug chloroquine accumulates in lysosomes and alters normal lysosomal processes, the effects of chloroquine on the lysosomal enzyme arylsulphatase B (ASB, N-acetylgalactosamine-4-sulphatase), which removes 4-sulphate groups from chondroitin-4-sulphate, were addressed. The underlying hypothesis derived from the recognized impairment of attachment of parasite-infected erythrocytes in the placenta, when chondroitin-4-sulphation was increased. If chloroquine reduced ASB activity, leading to increased chondroitin-4-sulphation, it was hypothesized that the anti-malarial mechanism of chloroquine might derive, at least in part, from suppression of ASB.

**Methods:**

Experimental methods involved cell culture of human placental, bronchial epithelial, and cerebrovascular cells, and the *in vitro *exposure of the cells to chloroquine at increasing concentrations and durations. Measurements of arylsulphatase B enzymatic activity, total sulphated glycosaminoglycans (sGAG), and chondroitin-4-sulphate (C4S) were performed using *in vitro *assays, following exposure to chloroquine and in untreated cell preparations. Fluorescent immunostaining of ASB was performed to determine the effect of chloroquine on cellular ASB content and localization. Mass spectrometry and high performance liquid chromatography were performed to document and to quantify the changes in chondroitin disaccharides following chloroquine exposure.

**Results:**

In the human placental, bronchial epithelial, and cerebrovascular cells, exposure to increasing concentrations of chloroquine was associated with reduced ASB activity and with increased concentrations of sGAG, largely attributable to increased C4S. The study data demonstrated: 1) decline in ASB activity following chloroquine exposure; 2) inverse correlation between ASB activity and C4S content; 3) increased content of chondroitin-4-sulphate disaccharides following chloroquine exposure; and 4) decline in extent of chloroquine-induced ASB reduction with lower baseline ASB activity. Confocal microscopy demonstrated the presence of ASB along the cell periphery, indicating extra-lysosomal localization.

**Conclusions:**

The study data indicate that the therapeutic mechanism of chloroquine action may be attributable, at least in part, to reduction of ASB activity, leading to increased chondroitin-4-sulphation in human placental, bronchial epithelial, and cerebrovascular cells. In vivo, increased chondroitin-4-sulphation may reduce the attachment of *P. falciparum*-infected erythrocytes to human cells. Extra-lysosomal localization of ASB and reduced impact of chloroquine when baseline ASB activity is less suggest possible mechanisms of resistance to the effects of chloroquine.

## Background

The presence of the sulphated glycosaminoglycan (sGAG), chondroitin-4-sulphate (C4S) has been demonstrated to affect the adherence of *Plasmodium falciparum*-infected erythrocytes to endothelial cells of the vasculature in models of malaria [1-6]. Attachment of the *P. falciparum*-infected erythrocytes was greater when sulphation of chondroitin-4-sulphate (C4S) was less, and increased sulphation of C4S reduced attachment of infected erythrocytes. Also, severity of malarial infection has been associated with the extent of sulphation of chondroitin-4-sulphate of the intervillous cells of the placenta in models of maternal-fetal transmission of malaria [7-12]. Since the accumulation of infected erythrocytes induces the capillary damage and organ dysfunction pathognomonic of malaria, consideration of the possible role of the enzyme arylsulphatase B (ASB; N-acetylgalactosamine-4-sulphatase), which hydrolyzes the 4-sulphate group of C4S, was of interest.

In humans, the enzyme ASB has been regarded as solely a lysosomal enzyme, since inborn deficiency of ASB causes the lysosomal storage disease Mucopolysaccharidosis VI (MPS VI), also known as Maroteaux-Lamy syndrome. In MPS VI, the accumulation of sulphated glycosaminoglycans, including C4S and dermatan sulphate, produces severe organ dysfunction and premature death. Significant effects of the enzyme ASB on the content of C4S in human colonic, bronchial, and mammary epithelial cells in tissue culture [13-16] were reported recently. These reports demonstrated extra-lysosomal localization of ASB in human colonic and bronchial epithelial cell, as well as other significant biological effects of ASB, affecting cell signaling, cell migration, and inflammation [13-17].

Since ASB activity affects the sulphation of C4S and sulphation of C4S affects the attachment of *P. falciparum*-infected red blood cells, modifications of ASB activity might influence malarial infectivity. If the lysosomotropic anti-malarial drug chloroquine could affect the activity of the enzyme ASB, this might influence disease activity and might explain, at least to some extent, the anti-malarial effect of chloroquine. In this report, evidence of the effect of chloroquine on ASB activity and C4S content in human placental, bronchial epithelial, and cerebrovascular cells in tissue culture is presented.

## Methods

### Cells and cell culture

Human placental fibroblastic (ATCC; Manassas, VA, USA), bronchial epithelial (C38 and IB3-1; ATCC), primary bronchial epithelial (BEC; Clonetics^® ^NHBE, Lonza, Walkersville, MD, USA), and primary cerebrovascular cells [CVC; ScienCellTM HBMEC (human brain microvascular endothelial cells), Carlsbad, CA] were obtained and grown according to recommendations and as previously reported [13-15]. IB3-1 and C38 cells were grown in 75 ml flasks coated with human fibronectin (10 μg/ml, Sigma-Aldrich, St. Louis, MO), vitrogen (30 μg/ml, Cohesion), and bovine serum albumin (10 μg/ml), using LHC-8 medium without phenol red (Invitrogen, Carlsbad, CA), containing 0.5 ng/ml recombinant epidermal growth factor, 6 mM glutamine, 0.005 mg/ml insulin, 500 ng/ml hydrocortisone, 0.035 mg/ml bovine pituitary extract, and 50 μg/ml gentamicin. Primary bronchial epithelial cells (BEC) were grown in BEGM^® ^(Clonetics). Cerebrovascular cells were grown in ECM (ScienCell), and placental cells were grown in DMEM with 10% FBS. All cells were grown in a 5% CO2 environment at 37°C and 95% humidity. Cells reached 80% confluence in about one week and were harvested by scraping, or by recommended EDTA-trypsin subculture reagent. Cell preparations were processed for measurements of ASB activity, ASB protein and mRNA expression, cell protein, sGAG, and C4S in control samples and following treatment with chloroquine (Sigma Chemical Company, St. Louis, MO) for 24 hours at a concentration of 50 nM, unless stated otherwise. CVC cells stained positively for CD31 using a standard protocol [Santa Cruz Biotechnology (SCBT), Santa Cruz, CA].

### Measurement of ASB activity

ASB measurements were by a fluorometric assay using the synthetic substrate 4-methylumbilliferyl sulphate (4-MUS), following a standard protocol [13-17]. Protein content of the cell homogenate was measured using BCA™ Protein Assay Kit (Pierce), and ASB activity was expressed as nmol/mg cell protein/hr. ASB protein in the control and chloroquine-treated cells was measured by a FACE-ELISA (fast-activated cell-based ELISA; Active Motif, CA). Placental cells were grown in a 96-well plate and treated with chloroquine 50 nM × 24 hours). Treated and control cells were washed and fixed with 4% formaldehyde in PBS. After 20 minutes, formaldehyde was aspirated, cells were washed with PBS, and treated with quenching buffer, to suppress endogenous peroxidase activity. Cells were treated with blocking buffer and incubated overnight at 4°C with polyclonal rabbit antibody to ASB (1:50; Open Biosystems, Huntsville, AL). Binding of primary antibodies to cells was detected by secondary goat anti-rabbit IgG-HRP, and bound HRP activity was developed by hydrogen peroxide/TMB substrate. The reaction was stopped by 2N sulfuric acid, and the optical density was measured in an ELISA plate reader (FLUOstar, BMG Labtech, Cary, NC) at 450 nm. After the reading, cells were washed thoroughly and incubated in crystal violet solution for 30 minutes, followed by 1% SDS (sodium dodecyl sulfate) solution for one hour. Colour was proportionate to the cell number which was measured by optical density using the ELISA plate reader at 592 nm. This reading was used to normalize the first reading (per equal number of cells), and the result was expressed as % of control for the ASB protein.

### Measurement of sulphated glycosaminoglycans

Total sulphated glycosaminoglycan (sGAG) content, including chondroitin-4-sulphate (C4S), chondroitin-6-sulphate (C6S), keratan sulphate, dermatan sulphate, heparan sulphate, and heparin, was measured in cell lysates by sulphated GAG assay (Blyscan™, Biocolor Ltd, Newtownabbey, Northern Ireland), using labeled 1,9-dimethylmethylene blue, as previously reported [14-16]. Hyaluronan and degraded disaccharide fragments were not measured by this assay. The reaction was performed in the presence of excess unbound dye. The cationic dye and GAG at acid pH produced an insoluble dye-GAG complex, and the GAG content was determined by the amount of dye that was recovered from the test sample following exposure to Blyscan Dissociation Reagent. Absorbance maximum of 1,9-dimethylmethylene blue is 656 nm. Concentration is expressed as μg/mg protein of cell lysate. Dye-GAG complex is proportional (1:1) to the sulphate content for C4S.

### Immunoprecipitation of cell lysates by chondroitin-4-sulphate

Cell lysates were prepared from chloroquine-treated and control cells. Chondroitin-4-sulphate (C4S; 4D1) antibody to native C4S was procured (SCBT). No prior enzymatic digestion of the cell samples by chondroitinase ABC was performed. Antibody was added to the cell lysates in tubes (C4S antibody at a concentration of 1 μg/mg protein; CS to a final dilution of 1:50), and the tubes were rotated overnight in a shaker at 4°C, as previously reported [14]. Next, 100 μl of pre-washed Protein L-Agarose (Santa Cruz) was added to each tube, tubes were incubated overnight at 4°C, and the Protein L-Agarose treated beads were washed three times with PBS containing Protease Inhibitor Cocktail. The precipitate was eluted with dye-free elution buffer and subjected to sulphated GAG assay.

### ASB Knockdown by siRNA

Small interfering RNA (siRNA) to knockdown ASB (NM_000046) was obtained commercially (Qiagen, Valencia, CA) and tested for effective knockdown by Western blot and by ASB activity assay. siRNA for ASB (150 ng; 0.6 μl) in 100 μl serum-free culture medium and 12 μl of HiPerfect Transfection Reagent (Qiagen) were combined, as previously described [14-16]. For ASB silencing, the siRNA sequences were:

Sense: 5'-GGGUAUGGUCUCUAGGCAtt-3'

Antisense: 5'-UUGCCUAGAGACCAUACCCtt-3'

For the negative control, the siRNA sequences were:

Sense: 5'-UUCUCCGAACGUGUCACGUtt-3'

Antisense: 5'-ACGUGACACGUUCGGAGAAtt-3'

### Confocal microscopy

For confocal microscopy, the BEC or CVC were grown in four-chamber tissue culture slides for 24 hours, as previously described [16]. Half of the wells were treated with chloroquine 50 nM for 24 hours; cells were then fixed in 95% ethanol for 30 min and refrigerated. Slides were washed once in 1× PBS containing 1 mM calcium chloride (pH 7.4) and permeabilized with 0.08% saponin. Preparations were washed with PBS, blocked with 5% normal goat serum, and incubated overnight with ASB polyclonal antibody (Open Biosystems, ThermoFisher Scientific, Hunstville, AL) at 4°C. Specificity of the ASB antibody was determined by inhibition of positive staining with the peptide that was the epitope for generation of the antibody. Slides were washed and stained with goat-anti-rabbit Alexa Fluor^® ^IgG 568 (1:100, Invitrogen) and with Alexa Fluor^® ^Phalloidin 488 (1:60, Invitrogen) to stain actin in the CVC cell preparations. Slides were washed thoroughly, and stained cells were observed with a Zeiss LSM 510 laser scanning confocal microscope (excitation 488 nm and 534 nm from an Ar/Kr laser. Green (F-actin) and red (ASB) fluorescence were detected through LP505 and 585 filters. The fluorochromes were scanned sequentially, and the collected images were exported by Zeiss LSM Image Browser software as TIFF files for analysis and reproduction.

### QPCR for ASB mRNA expression

C38 and IB3-1 lung epithelial cells were grown in 12-well tissue culture plates and treated with chloroquine 50 μM for 24 hours. At the end of the treatment, total RNA was isolated by RNeasy mini kit (Qiagen) from three control and three treated cell preparations. Three aliquots of equal amounts of RNA from each control or chloroquine-treated cell preparation were reverse transcribed and then amplified for 40 cycles, using Brilliant SYBR Green QRT-PCR MasterMix kit (Stratagene) and Mx3000P QRT-PCR system (Stratagene). ASB mRNA expression in control and treated cells was determined by amplification with the following primers for ASB (NCBI NM_000046), that were designed using Primer 3 software:

ASB forward: 5'AGACTTTGGCAGGGGGTAAT-3'

ASB reverse: 5'CAGCCAGTCAGAGATGTGGA-3'

Human β-actin was used an internal control. Fold changes in ASB mRNA expression due to chloroquine treatment were determined from the differences between the cycle thresholds using the following formulae and CT values that were the mean of nine determinations:

Fold change = 2^Δ3^ with Δ3 = Δ1 - Δ2, where Δ1 = CT control ASB - CT control actin and Δ2 = CT treated ASB - CT treated actin

### Isolation and purification of GAGs from cells

Cell samples were freeze dried and defatted by washing the samples with chloroform/methanol mixture of (2:1, 1:1, 1:2 (v/v)) each left overnight. The defatted samples (in 1 ml water) were individually subjected to proteolysis at 55°C with 10% of actinase E (20 mg/ml; Kaken Biochemicals, Tokyo, Japan) for 18 h. After proteolysis, dry urea and dry CHAPS were added to each sample [2 wt % (%, w/v) in CHAPS and 8 M in urea]. Particulates were removed from the resulting solutions by passing each through a syringe filter containing a 0.22 μm membrane. A Vivapure MINI Q H spin column [Viva Science (Edgewood, NJ)] was prepared by equilibrating with 0.5 ml of 8 M urea containing 2% CHAPS (pH 8.3). The clarified, filtered samples were loaded onto and run through the Vivapure MINI QH spin columns under centrifugal force (1000 × *g*). The columns were first washed with 0.5 ml of 8 M urea containing 2% CHAPS at pH 8.3. The columns were then washed five times with 0.5 ml of 200 mM NaCl. GAGs were released from the spin column by washing 1 time with 0.5 ml of 16% NaCl. Methanol (2 ml) was added to afford an 80 vol% (%, v/v) solution and the mixture was equilibrated at 4°C for 18 h. The resulting precipitate was recovered by centrifugation (2500 × *g*) for 15 min. The precipitate was recovered by dissolving in 1.0 ml of water. GAG solution was desalted using spin membrane (YM-3, 3000 MWCO, Millipore, Bedford, MA). GAGs were recovered and dissolved in 0.5 ml of water, and the recovered GAGs were stored frozen for further analysis.

### Enzymatic depolymerization of GAGs

GAG samples were incubated with chondroitinase ABC (10 milliunits; Seikagaku, Tokyo, Japan) and chondroitinase ACII (5 milli-units Seikagaku Corporation, Tokyo, Japan) at 37°C for 10 h. The enzymatic products were recovered by the centrifugal filtration (YM-3, Millipore). CS/DS disaccharides passed through the filter, were freeze-dried and ready for LC-MS analysis.

### Disaccharide composition analysis using liquid chromatography-mass spectrometry (LC-MS)

Unsaturated disaccharide standards of CS/DS (ΔDi-0S ΔUA-GalNAc, ΔDi-4S ΔUA-GalNAc4S, ΔDi-6S ΔUA-GalNAc6S, ΔDi-UA2S ΔUA2S-GalNAc, ΔDi-diSB ΔUA2S-GalNAc4S, ΔDi-diSD ΔUA2S-GalNAc6S, ΔDi-diSE ΔUA-GalNAc4S6S, ΔDi-triS ΔUA2S-GalNAc4S6S) were obtained (Seikagaku).

The LC-MS analysis was performed on a LC-MS system (Agilent, LC/MSD trap MS). Solutions A and B for HPLC were 0% and 75% acetonitrile, respectively, containing the same concentration of 15 mM Hexylamine (HXA) and 100 mM 1,1,1,3,3,3,-hexafluoro 2-propanol (HFIP). The flow rate was 100 μl/min. The separation was performed on an ACQUITY UPLC^TM^ BEH C18 column (Waters, 2.1 × 150 mm, 1.7 μm) at 45°C using solution A for 10 min, followed by a linear gradient from 10 to 40 min of 0% to 50% solution B. The column effluent entered the source of the ESI-MS for continuous detection by MS. The electrospray interface was set in positive ionization mode with the skimmer potential 40.0 V, capillary exit 40.0 V and a source of temperature of 350°C to obtain maximum abundance of the ions in a full scan spectra (150-1500 Da, 10 full scans/s). Nitrogen was used as a drying (8 liters/min) and nebulizing gas (40 p.s.i.). The data were collected by UV and mass, respectively. Extracted ion chromatography (EIC) was processed using Bruck software.

### Statistical analysis

Statistical significance of the data was analyzed using Instat software. Unless stated otherwise, one-way ANOVA with Tukey-Kramer post-test was performed with p < 0.05 considered significant. At least three independent experiments were performed for each analysis, and technical replicates of each sample were performed. In the figures, *** represents p < 0.001, ** p < 0.01, and * p < 0.05.

## Results

### Chloroquine reduces ASB activity

**Figure 1:**
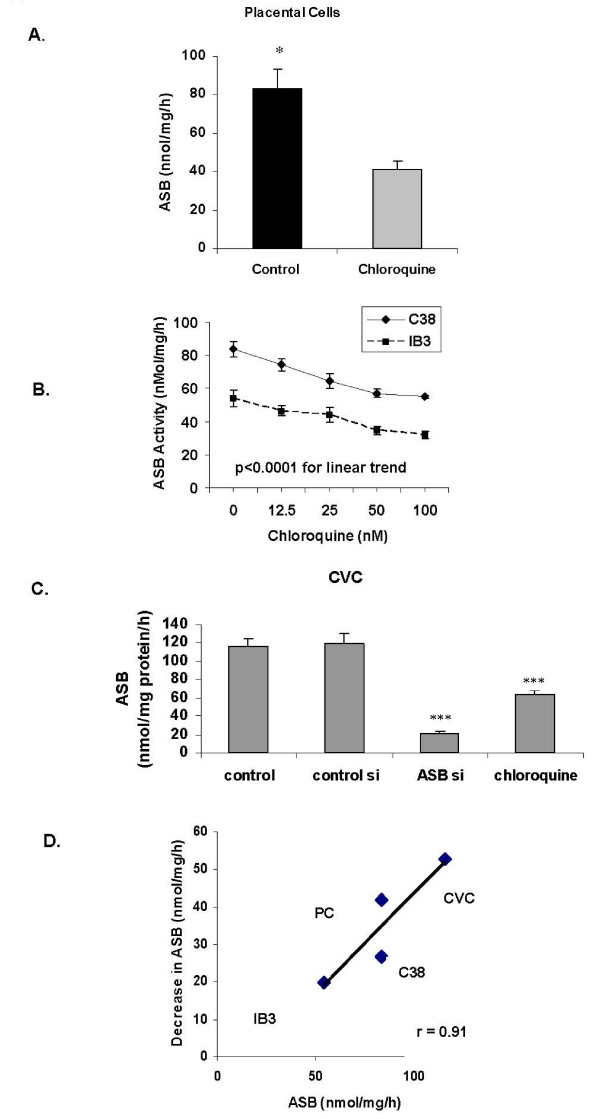
**ASB activity declines following exposure to chloroquine (CQ)**. ASB activity declined in the placental fibroblasts following chloroquine exposure (**Panel A**; p = 0.02, unpaired t-test, two-tailed). ASB activity was significantly higher in the C38 than in the IB3-1 cells. Following exposure to increasing concentrations of chloroquine, the ASB activity declined linearly in both cell lines (**Panel B**; p < 0.0001, 1-way ANOVA for linear trend; r^2 ^= 0.89 for C38 and r^2 ^= 0.86 for IB3-1). In the CVC, chloroquine exposure produced significant reduction in ASB activity (**Panel C**; p < 0.001, 1-way ANOVA with Tukey-Kramer post-test). Following ASB silencing by siRNA, the ASB activity was reduced more than by chloroquine. The extent of reduction in ASB activity is less when the baseline ASB activity is less, as demonstrated by the direct correlation between the change in ASB activity and the baseline ASB activity (**Panel D**; r = 0.91). [con si = control siRNA; ASB si = ASB siRNA; CVC = cerebrovascular cells; PC = placental cells; ASB = arylsulphatase B].

ASB activity post exposure to chloroquine (50 nM × 24 hours) was measured in the human placental, bronchial epithelial, and CVC cells. In the placental cells, ASB activity post-chloroquine exposure declined by ~50%, from a baseline of 83.5 ± 9.8 nmol/mg/h to 41.6 ± 3.6 nmol/mg/h (Figure 1A). In the C38 and IB3-1 cells, progressive decline in ASB activity followed exposure to increasing concentrations of chloroquine (Figure 1B; p < 0.0001 for linear trend). When ASB was silenced by siRNA in the CVC cells, the decline in ASB activity was greater than the decline following chloroquine, but both were significant (Figure 1C; p < 0.001, 1-way ANOVA with Tukey-Kramer post-test). The decline in ASB activity following chloroquine was proportional to the baseline activity, with greatest decline in the CVC that had the highest initial level (Figure 1D).

### Chloroquine increases total sulphated glycosaminoglycans (sGAG) and chondroitin-4-sulphate (C4S)

**Figure 2:**
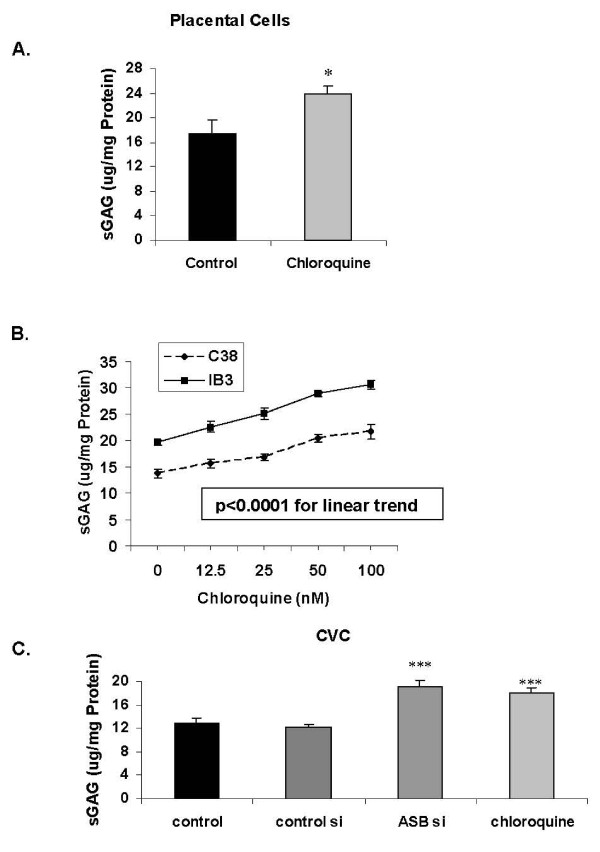
**Increased total sulphated glycosaminoglycan (sGAG) content following chloroquine exposure**. In the placental cells, the sGAG content increased significantly following chloroquine (50 nM × 24 hours) (**Panel A**; p = 0.02, unpaired t-test, two-tailed). Similarly, the sGAG content increased linearly in response to exposure to increasing concentrations of chloroquine (**Panel B**; p < 0.0001, 1-way ANOVA for linear trend; r^2 ^= 0.92 for C38 cells and r^2 ^= 0.96 for IB3-1 cells). For the CVC, the increase in sGAG was significant following chloroquine (50 nM × 24 hours) and ASB siRNA (× 24 hours) (**Panel C**; p < 0.001, 1-way ANOVA with Tukey-Kramer post-test). [con si = control siRNA; ASB si = ASB siRNA; CVC = cerebrovascular cells; PC = placental cells; ASB = arylsulphatase B].

**Figure 3:**
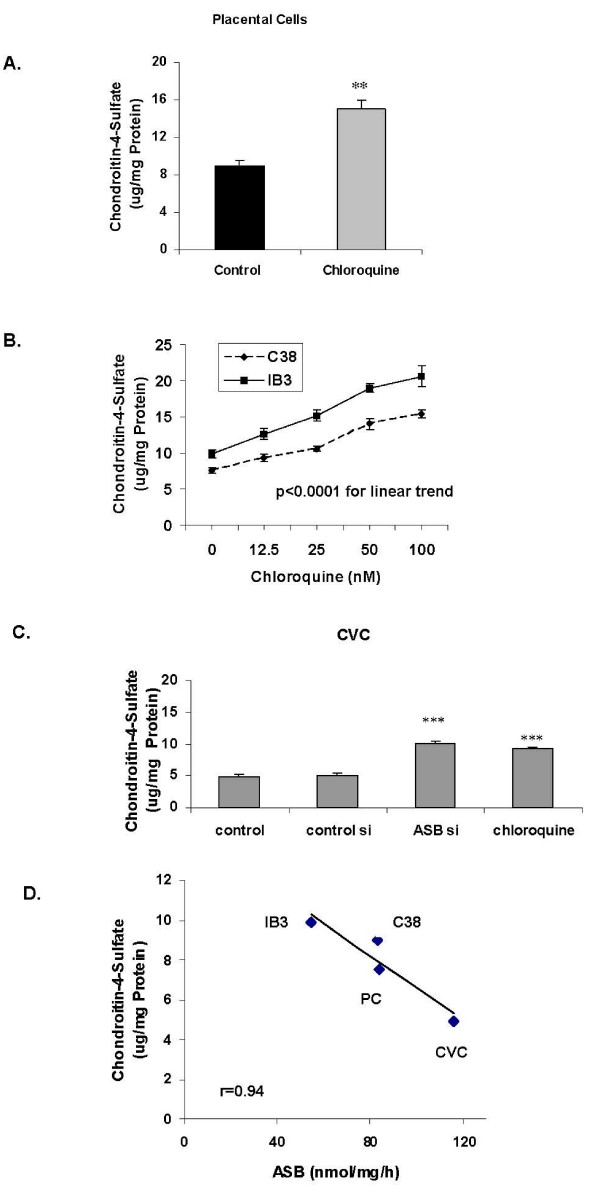
**Increased chondroitin-4-sulphate (C4S) content following chloroquine exposure**. The concentration of C4S increased significantly following exposure to increasing concentrations of chloroquine in the placental cells for 24 hours (**Panel A**; p = 0.002, unpaired t-test, two-tailed) and in the C38 and IB3-1 cells for 24 hours (**Panel B**; p < 0.0001, 1-way ANOVA for linear trend; r^2 ^= 0.96 for C38 cells and r^2 ^= 0.96 for IB3-1 cells). For the CVC cells, the increase in C4S was significant following chloroquine (50 nM × 24 hours) and ASB siRNA (× 24 hours) (**Panel C**; p < 0.001, 1-way ANOVA with Tukey-Kramer post-test). The decline in ASB activity was inversely correlated with the increase in C4S content (**Panel D**; r = 0.94). [con si = control siRNA; ASB si = ASB siRNA; CVC = cerebrovascular cells; PC = placental cells].

Marked increase in sGAG followed exposure to chloroquine in the placental, IB3-1, C38, and CVC cells (Figures 2A, B, C), consistent with reduced ASB activity leading to accumulation of sGAG. When cell lysates were immunoprecipitated with the C4S antibody and the resulting sGAG measured by the Blyscan assay (Figures 3A, B, C), it was apparent that the increase in sGAG was largely attributable to increase in C4S, and was consistent with a specific effect on reduction of ASB activity by chloroquine. The increases in sGAG and C4S with increasing concentrations of chloroquine showed a linear trend in the C38 and IB3-1 cells (Figure 3B; p < 0.0001). In the CVC, increases in sGAGs and C4S following chloroquine were somewhat less than following ASB silencing, but both were significant (Figure 3C; p < 0.001, 1-way ANOVA with Tukey-Kramer post-test). ASB activity was inversely correlated with C4S content in the four cell lines tested (Figure 3D).

### Disaccharide composition of CVC, IB3-1 and C38 cells

**Figure 4:**
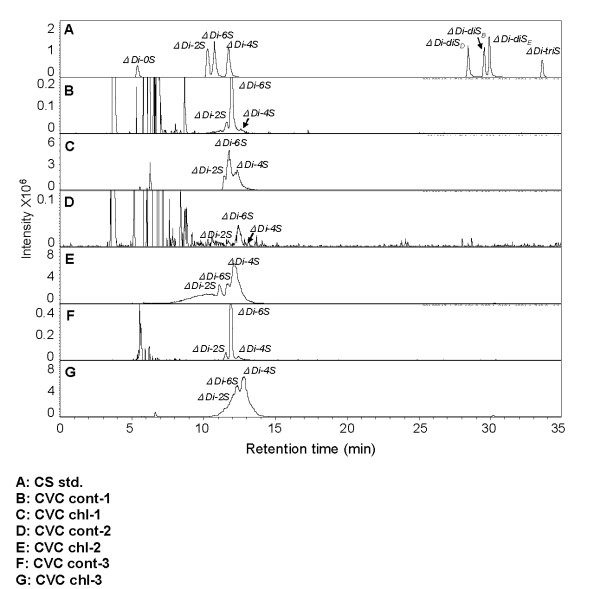
**Extracted ion chromatography (EIC) representation of disaccharide composition in cerebrovascular cells before and after exposure to chloroquine**. The HPLC system used resolves the eight CS disaccharide standards (**Panel A**). Despite the variability of retention time due to variable quantities of residual buffer salts, peak identity was easily confirmed by co-injection with single known CS disaccharide standards as well as from their MS (see Additional File 1) or by MS/MS spectra. CVC controls (**Panels B, D, and F**) and CVC after chloroquine treatment (**Panels C, E, and G**) are shown. The chloroquine-treated CVC demonstrate marked increase in the peak for C4S disaccharide (ΔDi-4S) following chloroquine exposure (**Panels E and G vs. Panel F**). Additional peaks observed early (3-8 min) in the chromatograms B, D, and F and well separated from the sulpho-group containing CS standards are residual contaminants from cells and media and do not interfere with the analysis.

Disaccharide composition of treated and control CVC is listed in Table 1. Because of partial peak overlap, quantification relied on MS using a method previously demonstrated for heparan sulfate disaccharide analysis [18]. Figure 4 (also see Additional File 1) is extracted ion chromatography (EIC) of CS/DS disaccharide analysis of CVC cells. The percentage of C4S increased significantly in the CVC.

**Table 1:** CS/DS disaccharide composition of cerebrovascular cells*

Composition (%)	ΔDi-0S	ΔDi-UA2S	ΔDi-6S	ΔDi-4S	ΔDi-diSD	ΔDi-diS	ΔDi-diSE	ΔDi-triS
CVC-cont-1	n.d.	8.5	84.3	7.2	n.d.	n.d.	n.d.	n.d.
CVC-1	n.d.	10.3	43.9	45.2	0.22	0.08	0.3	n.d.
CVC-cont-2	n.d.	7	90	3	n.d.	n.d.	n.d.	n.d.
CVC-2	n.d.	17.3	16.2	66	0.09	0.02	0.39	n.d.
CVC-cont-3	n.d.	8.1	84.9	7	n.d.	n.d.	n.d.	n.d.
CVC-3	n.d.	24.1	22.9	52.2	0.12	0.03	0.65	n.d.

*CS = chondroitin sulphate; DS = dermatan sulphate; CVC = cerebrovascular cells; cont = control; CVC-1, CVC-2, CVC-3 = chloroquine-treated; n.d. = not detected

DDi-0S: 2-acetamido-2-deoxy-3-*O*-(b-D-*xylo*-hex-4-enopyranosyluronic acid)-D-galactose

DDi-4S: 2-acetamido-2-deoxy-4-*O*-sulphosulpho-3-*O*-(b-D-*xylo*-hex-4-enopyranosyluronic acid)-D-galactose

DDi-6S: 2-acetamido-2-deoxy-6-*O*-sulpho-3-*O*-(b-D-*xylo*-hex-4-enopyranosyluronic acid)-D-galactose

DDi-UA2S: 2-acetamido-2-deoxy-3-*O*-(2-*O*-sulpho-b-D-*xylo*-hex-4-enopyranosyluronic acid)-D-galactose

DDi-diSB: 2-acetamido-2-deoxy-4-*O*-sulpho-3-*O*-(2-*O*-sulpho-b-D-*xylo*-hex-4-enopyranosyluronicacid)-D-galactose

DDi-diSD: 2-acetamido-2-deoxy-6-*O*-sulpho-3-*O*-(2-*O*-sulpho-b-D-*xylo*-hex-4-enopyranosyluronicacid)-D-galactose

DDi-diSE: 2-acetamido-2-deoxy-4,6-di-*O*-sulpho-3-*O*-(b-D-*xylo*-hex-4-enopyranosyluronic acid)-D-galactose

DDi-triS: 2-acetamido-2-deoxy-4,6-di-*O*-sulpho-3-*O*-(2-*O*-sulpho-b-D-*xylo*-hex-4-enopyranosyluronic acid)-D-galactose acid)-D-galactose

The chondroitin sulphate content in the C38 and IB3-1 cells that were grown to confluency in a T75 flask was calculated by a quantitative method for HPLC. The peak areas of CS disaccharide standards in UV chromatography are functions of their amounts. Based on the linear relation between the UV peak areas, the amount of disaccharides in C38 and IB3-1 cells was calculated. In the C38 cells, the CS content was 70 μg and increased to 310 μg following chloroquine. In the IB3-1 cells, the chondroitin sulfate increased from 330 μg to 650 μg following chloroquine. In the C38 and IB3-1 cells, the percentage of C4S disaccharides varied less following chloroquine than in the CVC.

### Confocal imaging of ASB in CVC and bronchial epithelial cells

**Figure 5:**
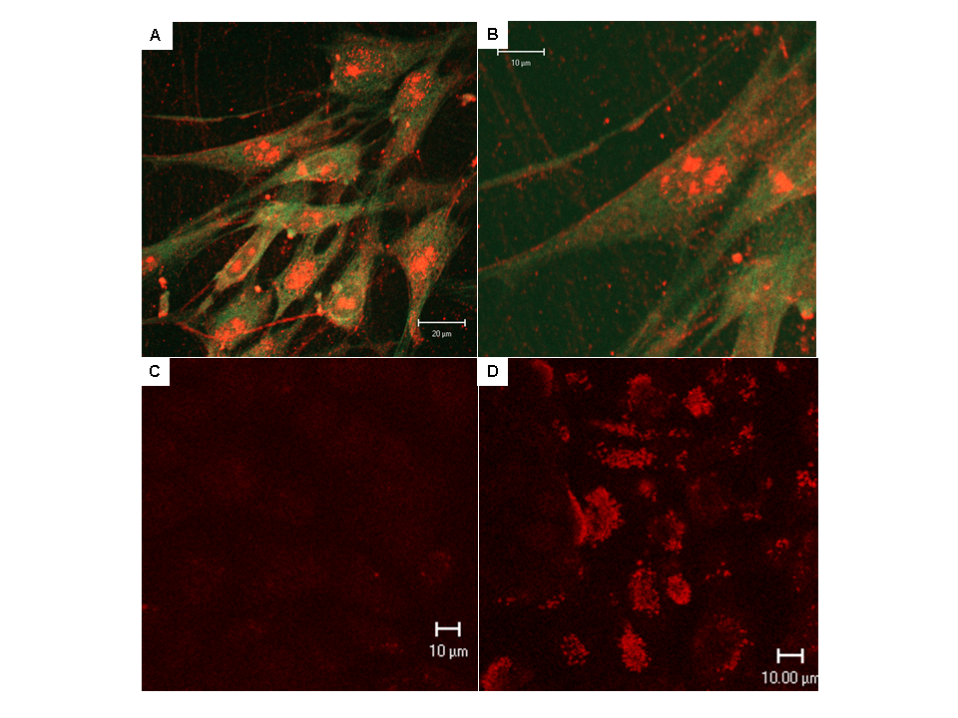
**Confocal microscopy demonstrates cell surface localization of ASB**. Confocal microscopy demonstrates the presence of peripheral localization of ASB in the CVC, as well as cytoplasmic and nuclear localization. ASB is stained red, and β-actin is stained green (**Panels A, B**). In primary bronchial epithelial cells, exposure to chloroquine (50 nM × 24 hours) produced marked reduction in ASB fluorescent immunostaining (**Panel C**), compared to untreated control (**Panel D**). [CVC = cerebrovascular cells; ASB = arylsulphatase B; BEC = bronchial epithelial cells].

Confocal images of ASB were obtained following fluorescent immunostaining, in which ASB appears red and β-actin appears green (Figure 5A, B). The presence of ASB was demonstrated along the cell periphery, consistent with membrane localization. Nuclear and cytoplasmic localization were also evident. The membrane localization was unexpected, since ASB had been regarded as solely a lysosomal enzyme. In the primary bronchial epithelial cells (BEC), fluorescent immunostaining for ASB was markedly reduced following exposure to chloroquine (Figure 5C) vs. untreated control (Figure 5D), and was evident along the cell periphery.

### ASB activity, ASB protein content, mRNA expression, and total sGAG

The ASB protein content in the placental cells was measured by a Fast Activated Cell-based ELISA (FACE) and compared with the ASB activity after exposure to chloroquine 50 nM for 24 hours. ASB activity declined from 83.5 ± 9.8 to 41.6 ± 3.6 nmol/mg protein/hr (to 49.8% of baseline), and ASB protein content declined to 47.6 ± 5.2% of the baseline, indicating the close correspondence between the activity measurement and the FACE assay.

**Figure 6:**
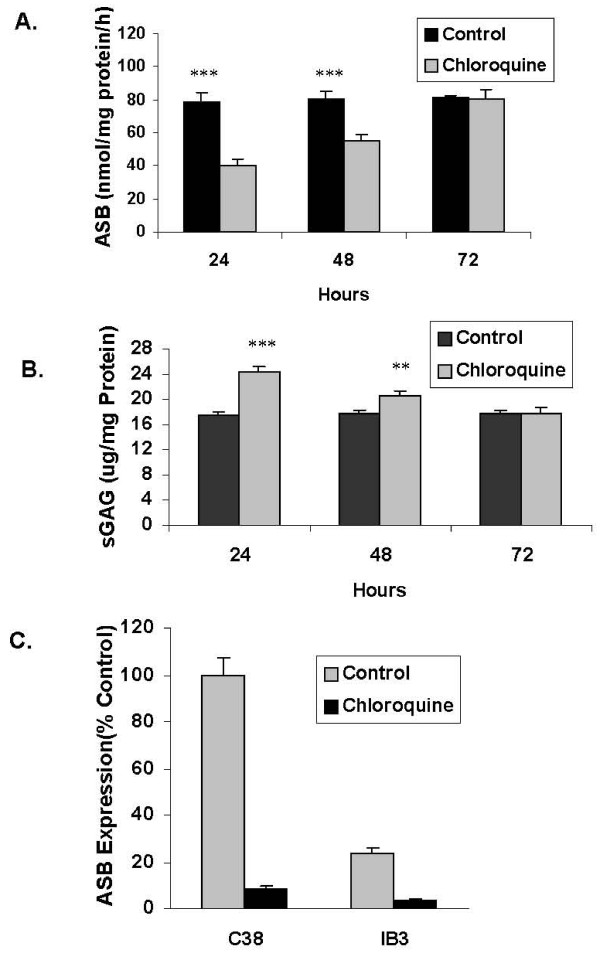
**Relationship between ASB activity and total sGAG following chloroquine exposure over 72 hours**. Chloroquine (50 nM) was administered for 24 hours, the spent media removed, and fresh media without chloroquine was supplied at 24 and 48 hours. ASB activity (**Panel A**) and total sGAG (**Panel B**) were measured in spent media at 24, 48, and 72 hours, as shown. ASB activity rose from the nadir at 24 hours and returned to baseline by 72 hours (p < 0.001 at 24 and 48 hours; 1-way ANOVA with Tukey-Kramer post-test). Inversely, the sGAG content peaked at 24 hours and returned to baseline by 72 hours (p < 0.001 at 24 hours, p < 0.01 at 48 hours; 1-way ANOVA with Tukey-Kramer post-test). QPCR demonstrated less baseline mRNA expression of ASB in the IB3-1 cells than in the C38 cells (**Panel C**), and declines in both cell lines following exposure to chloroquine (50 μM × 24 hours). Cycle threshold of ASB was calculated in comparison to cycle threshold of β-actin, which varied only from 29.36 to 30.27.

Placental cells were treated with chloroquine 50 nM × 24 hours and fresh media added at 24 and 48 hours. Spent media were collected at 24, 48, and 72 hours, and ASB activity (Figure 6A) and total sGAG were measured (Figure 6B). ASB activity nadir was at 24 hours, with return to baseline by 72 hours. Inversely, the sGAG content peaked at 24 hours and returned to baseline by 72 hours. These findings indicate that the effects of chloroquine exposure on ASB activity and sGAG content are prolonged beyond the time of direct exposure, but have dissipated by 72 hours.

Following chloroquine treatment in the C38 and IB3-1 cells, the mRNA expression of ASB declined significantly, in contrast to β-actin expression (Figure 6C). ASB cycle threshold (CT) in untreated C38 cells was 29.7 ± 0.2 compared to CT of 32.4 ± 0.4 in the chloroquine-treated cells, with β-actin values of 30.3 ± 0.9 and 29.4 ± 0.2. Similarly, in the IB3-1 cells, CT for ASB in control cells was 31.0 ± 0.2 and increased to 33.8 ± 1.3 following chloroquine exposure, with β-actin values of 29.5 ± 0.3 and 30.1 ± 0.4. These results indicate a 12-fold decrease in ASB mRNA expression in the C38 cells and a 5-fold decrease in the IB3-1 cells following chloroquine exposure.

## Discussion

The study findings indicate that exposure to chloroquine reduced the ASB activity and expression and, correspondingly, increased the content of chondroitin-4-sulphate, as well as total sGAGs, in human placental, bronchial, and cerebrovascular cells. C4S has been identified as the cell surface molecule associated with adherence of infected erythrocytes to *Saimiri *brain and human lung endothelial cells, as well as placental cells [2]. Since low-sulphated chondroitin sulphate has been implicated in the increased adherence of *P. falciparum*-infected red blood cells in the human placenta [7-12], the *in vitro *findings in this report may be relevant to the mechanism of clinical infection, particularly if placental infections are attributable to the expression of novel variant surface antigens that bind preferentially to C4S [19]. Reduced ASB activity and the associated increase in C4S sulphation in human vascular or bronchial cells post-chloroquine may hinder the attachment of parasite-infected erythrocytes *in vivo*.

The low-sulphated chondroitin sulphate proteoglycan receptors of placenta are found predominantly in the intervillous space, and parasite-infected red blood cells sequester in the human placenta through attachment to C4S. The major attachment to C4S is reported to be by the *Plasmodium falciparum *erythrocyte membrane protein 1 (PfEMP1), encoded by the *var2csa *gene [20].

Chloroquine has been found to accumulate in lysosomes, and by effects on lysosomal pH, its exit from the lysosome is prevented [21,22]. Since ASB is also found in the lysosome, chloroquine, by effects on lysosomal pH, may reduce the lysosomal ASB activity *in vivo*, since ASB functions optimally at low pH. The study finding that chloroquine exposure reduced the mRNA expression of ASB, as well as the ASB protein and activity in the *in vitro *assay, indicates that chloroquine directly affects mRNA content. Study data (not shown) demonstrated that activity of the lysosomal enzyme galactose-6-sulphatase (GALNS) also declined post-chloroquine exposure in the CVC and placental cells, although the decline was not as much as that found in ASB activity.

Chloroquine has been used for treatment of malarial infections for many decades. The mechanism of action of chloroquine has been attributed to various effects, including entrapment in the parasite's food vacuole, inhibition of haem polymerase with accumulation of the toxic Fe(II)-protoporphyrin IX-chloroquine complex that disrupts membrane function and leads to parasite autodigestion, or interference with nucleic acid biosynthesis by intercalation with parasitic DNA [21-23]. The current study findings neither refute nor support these mechanisms, but suggest an additional biochemical mechanism to explain the effect of chloroquine.

The study data suggest that development of resistance to chloroquine may be, at least in part, attributable to the presence of extra-lysosomal ASB, which would be unaffected by chloroquine that is accumulated and sequestered in the lysosomes. Also, since the decline in ASB activity was less when the baseline ASB activity was less, the effectiveness of chloroquine might be expected to diminish as ASB activity declined.

Further attention to the effects of ASB on sulphation of C4S may facilitate development of innovative anti-malarial therapies. Improved understanding of the mechanism of action of chloroquine might provide new insight into mechanisms of resistance, leading to improved therapeutic approaches to what remains one of the most prevalent and deadly infectious diseases.

## Conclusions

Study data demonstrate that exposure of human placental, bronchial, and cerebrovascular cells in tissue culture to chloroquine leads to reduced mRNA and protein expression and activity of the enzyme arylsulphatase B, and to increased cellular content of chondroitin-4-sulphate, as well as total sulphated glycosaminoglycans. Since the degree of sulphation of chondroitin-4-sulphate has been associated with the attachment of *P. falciparum*-infected erythrocytes to the intervillous cells of the placenta, chloroquine-induced decline in ASB activity, with the accompanying increase in chondroitin-4-sulphation, may produce its anti-malarial effect. Extra-lysosomal ASB and the decline in further reduction of ASB activity at lower activity levels may contribute to development of resistance to chloroquine.

## Abbreviations

ASB: arylsulphatase B; BEC: bronchial epithelial cell; C4S: chondroitin-4-sulphate; CVC: cerebrovascular cell; CQ: chloroquine; CT: cycle threshold; EIC: extracted ion chromatography GAG: glycosaminoglycan; LC-MS: liquid chromatography-mass spectrometry; MPS: mucopolysaccharidosis; QPCR: quantitative polymerase chain reaction; sGAG: sulphated glycosaminoglycan; RBC: red blood cell; PfEMB1: *Plasmodium falciparum *erythrocyte membrane protein-1.

## Competing interests

The authors declare that they have no competing interests.

## Authors' contributions

SB planned experiments with JKT, performed the experiments, and prepared many of the figures. JKT planned experiments with SB, performed confocal microscopy, and prepared the manuscript. ZZ, RJL and KS performed the isolation and purification of the GAGs from the cells and the LC-MS analysis to determine the disaccharide composition of the GAGs. They wrote the sections of the manuscript, the table, and the figures that present these methods and results. All authors read and approved the final manuscript.

## Supplementary Material

Additional file 1 **Detailed mass spectrometry data of cerebrovascular cell disaccharides**. The file contains detailed analysis of the cerebrovascular cell disaccharides that were present following isolation and purification of the cellular sGAG, followed by their enzymatic depolymerization, and separation and detection by LC-MS, as detailed in the Methods. Figure 4A presents peaks of the disaccharide standards, including ΔDi-0S, ΔDi-UA2S, ΔDi-6S, ΔDi-4S, ΔDi-diSD, ΔDi-diSB, ΔDi-diSE, and ΔDi-triS. Figures 4B-4G present mass spectra for ΔDi-0S, ΔDi-UA2S, and ΔDi-6S, in the control (4B,4F) or chloroquine-treated (4C,4E,4G) cerebrovascular cells, with demonstrable changes in amplitude of the peaks for the disaccharides of major interest in this report. Click here for file
