# Supplementary figures and images for: Chloroquine reduces arylsulphatase B activity and increases chondroitin-4-sulphate: implications for mechanisms of action and resistance
Source: Malar J. 2009 Dec 17;8:303. doi: 10.1186/1475-2875-8-303 (PMC2805689; doi:10.1186/1475-2875-8-303)

Figure 4A

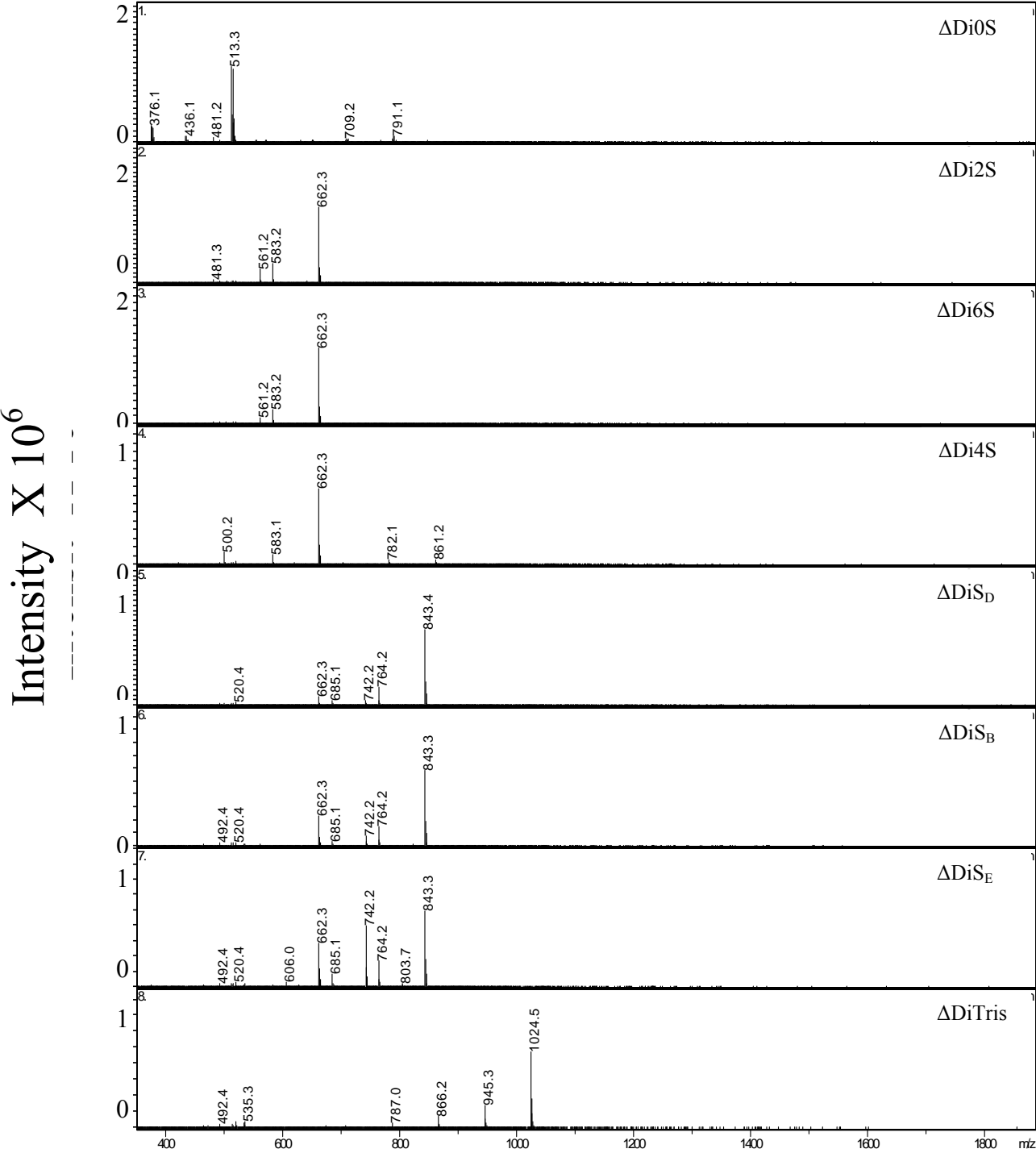

Figure 4B

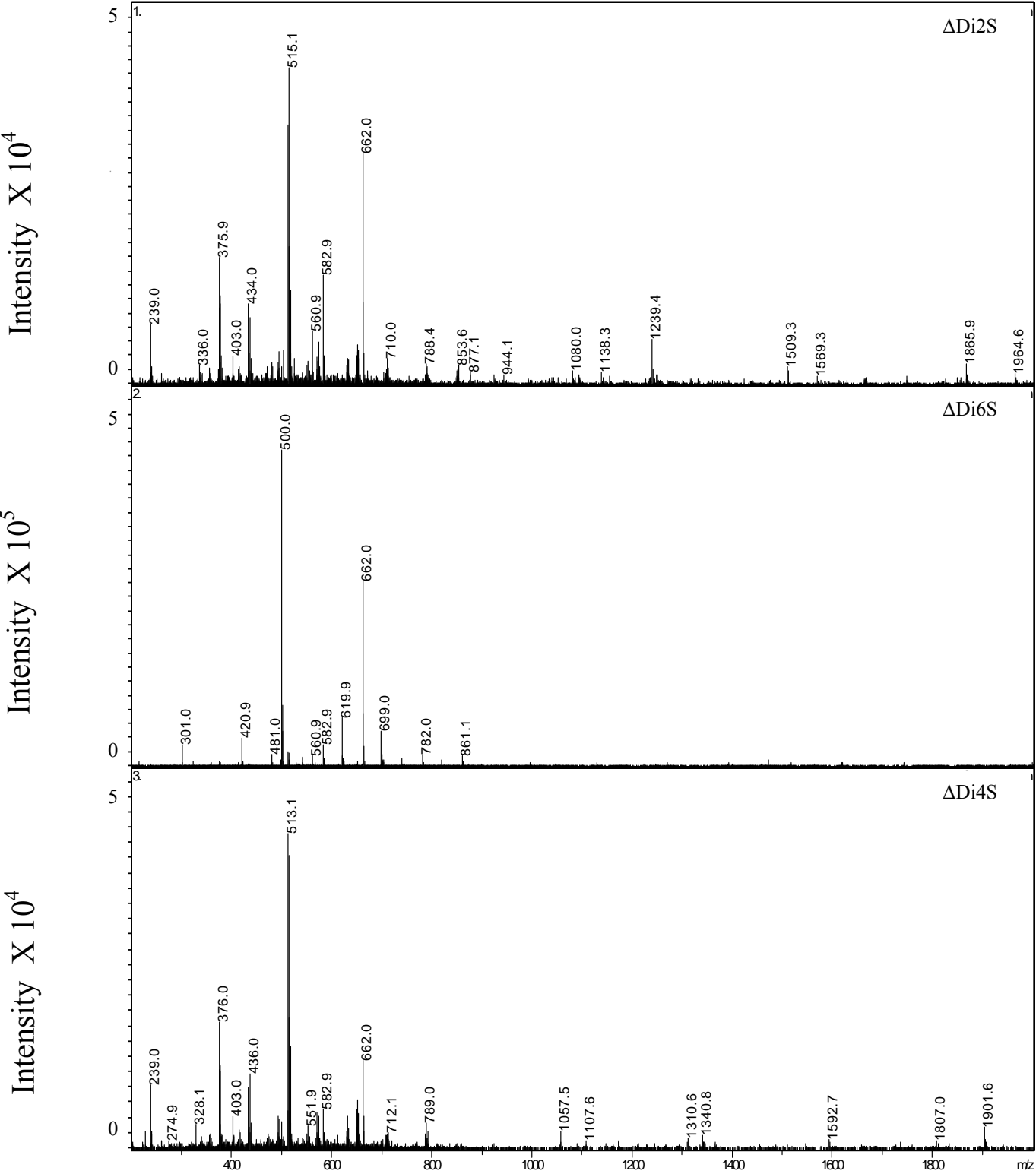

Figure 4C

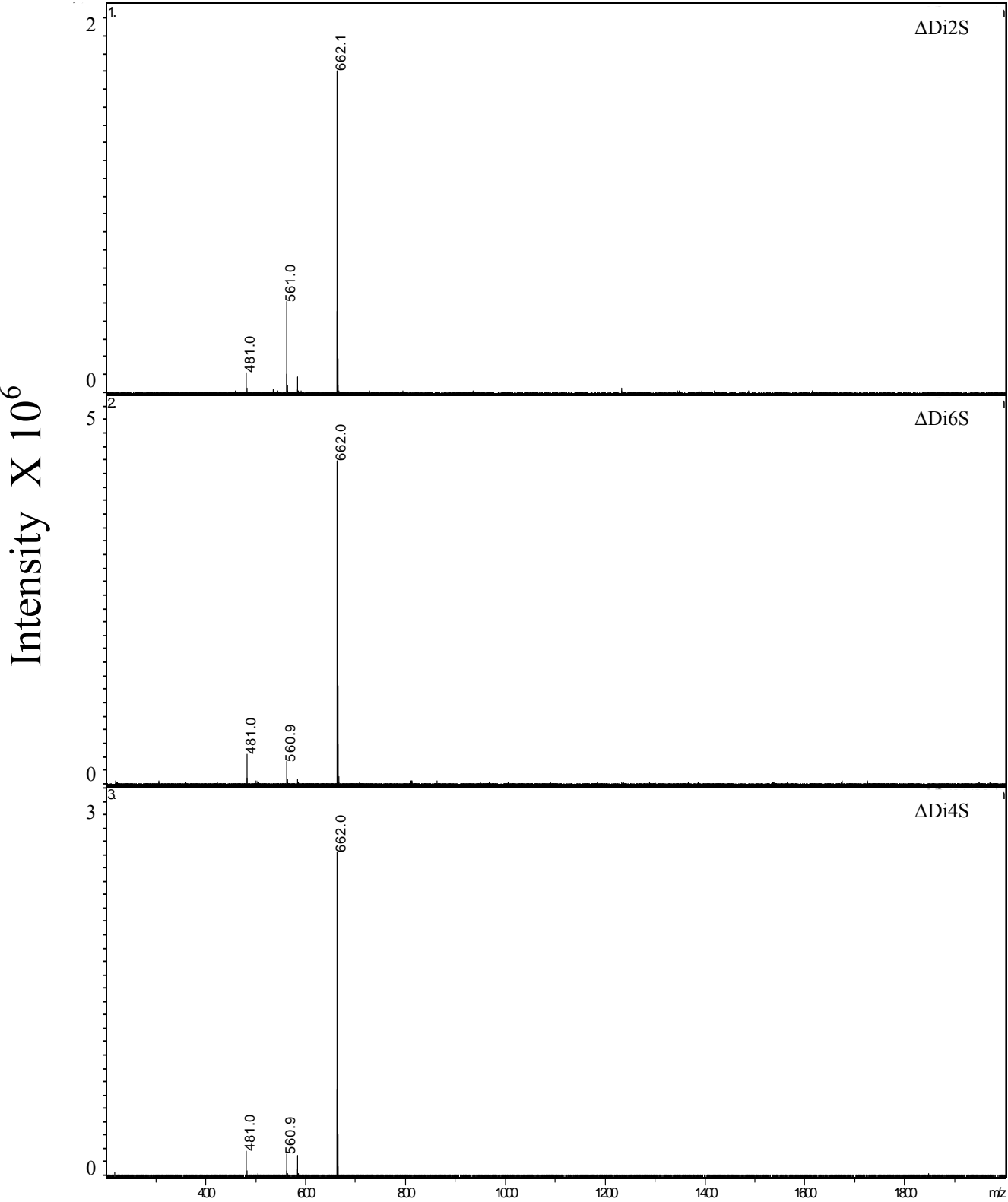

Figure 4E

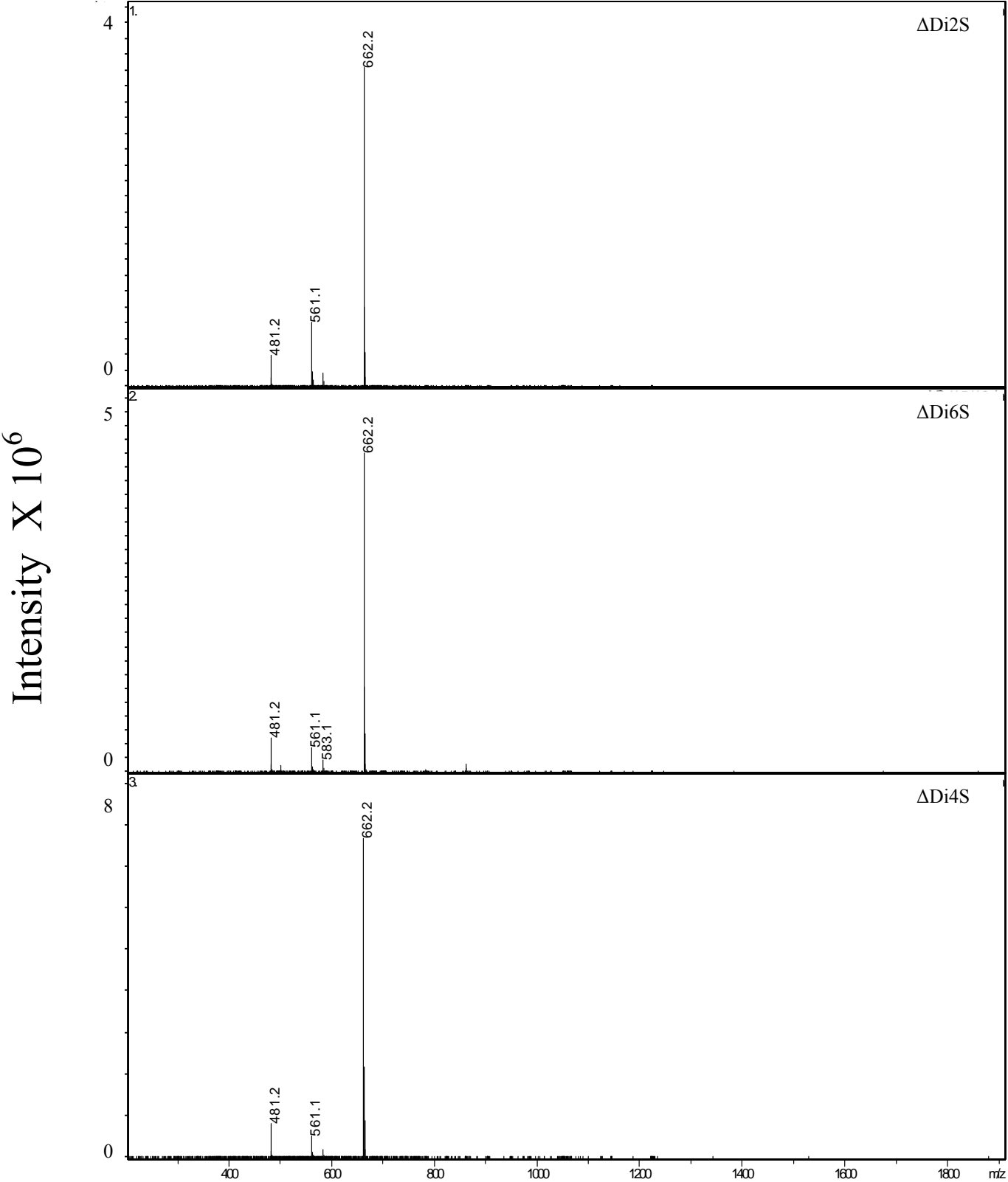

Figure 4F

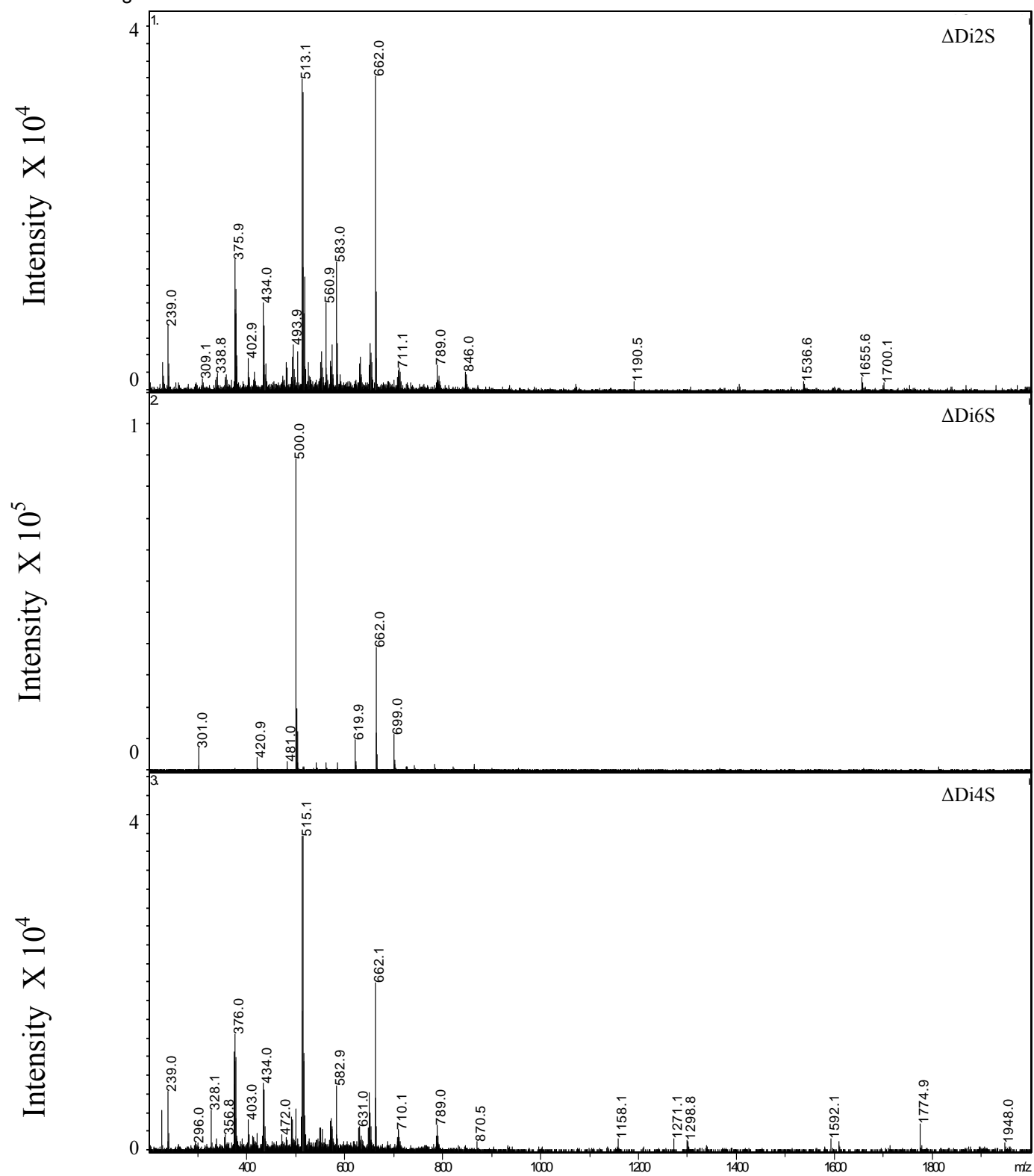

Figure 4G

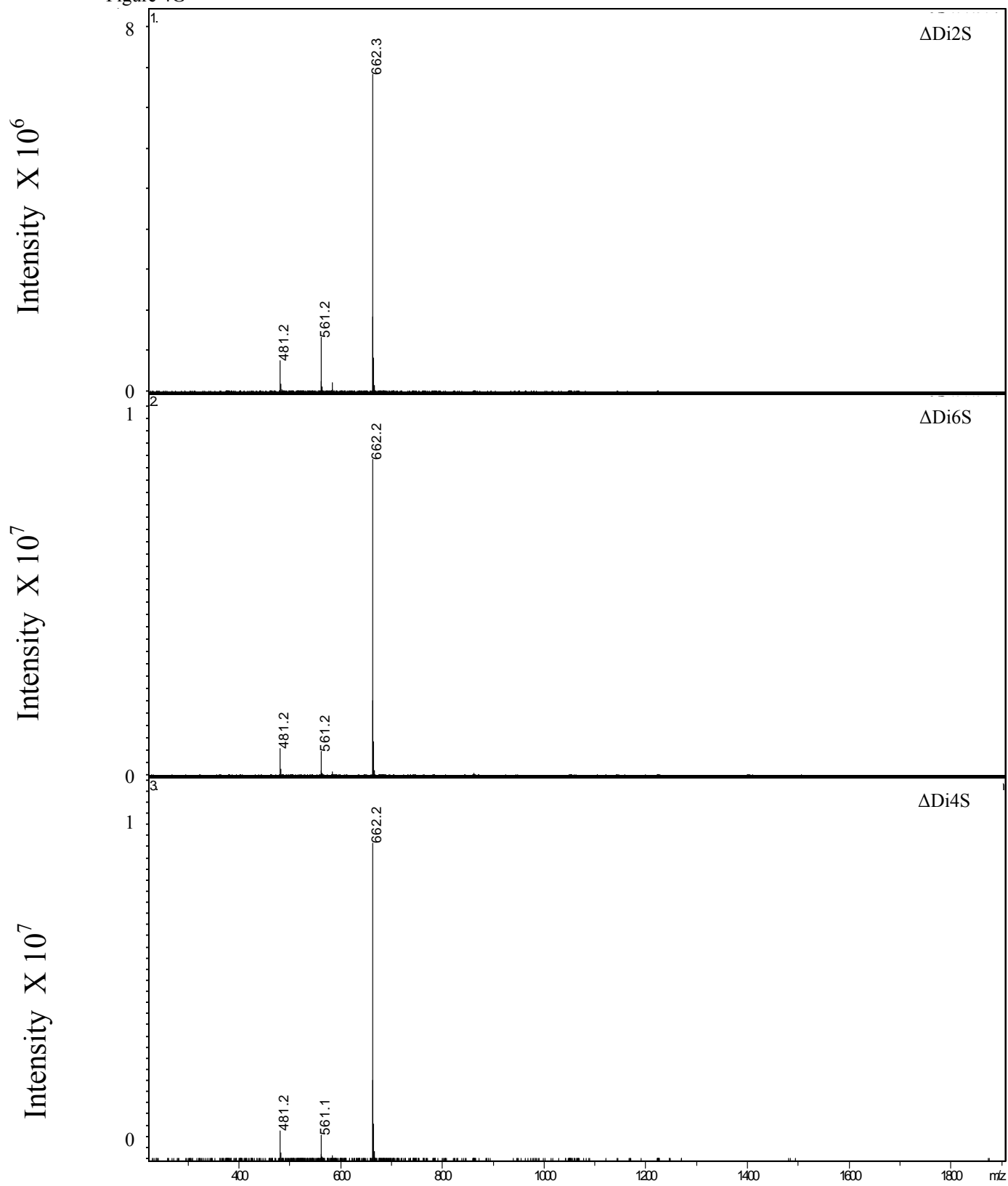

Supplement: Additional file 1 — Detailed mass spectrometry data of cerebrovascular cell disaccharides. The file contains detailed analysis of the cerebrovascular cell disaccharides that were present following isolation and purification of the cellular sGAG, followed by their enzymatic depolymerization, and separation and detection by LC-MS, as detailed in the Methods. Figure 4A presents peaks of the disaccharide standards, including ΔDi-0S, ΔDi-UA2S, ΔDi-6S, ΔDi-4S, ΔDi-diSD, ΔDi-diSB, ΔDi-diSE, and ΔDi-triS. Figures 4B-4G present mass spectra for ΔDi-0S, ΔDi-UA2S, and ΔDi-6S, in the control (4B,4F) or chloroquine-treated (4C,4E,4G) cerebrovascular cells, with demonstrable changes in amplitude of the peaks for the disaccharides of major interest in this report. [file 1475-2875-8-303-S1.PDF]
